# Bone Optimization for Perioperative Spine Patients: A Multidisciplinary Approach at a Single Academic Center

**DOI:** 10.3390/jcm14248866

**Published:** 2025-12-15

**Authors:** Maria Valentina Suarez-Nieto, Karen Malacon, Andrea Fox, Mary Carmen Lopez Isidro, Harsh Wadhwa, Serena S. Hu, Corinna C. Zygourakis

**Affiliations:** 1 Department of Neurosurgery, Stanford University School of Medicine, Stanford, CA 94305, USA; 2 Department of Orthopaedic Surgery, Stanford Health Care, Stanford, CA 94304, USA

**Keywords:** bone optimization, perioperative spine patient, fracture liaison service, bone health clinic

## Abstract

**Background**: Osteoporosis and low bone mineral density are highly prevalent among patients undergoing spinal fusion, contributing to higher rates of hardware failure, revision surgery, and poor postoperative outcomes. Despite clear risks, perioperative screening and treatment for osteoporosis remain inconsistent. Bone Health Clinics (BHCs) and Fracture Liaison Services (FLSs) have emerged as multidisciplinary models to address this care gap. We describe the implementation of a dedicated BHC at a single academic center and evaluate perioperative pharmacotherapy patterns, treatment barriers, and surgical outcomes among spine patients. **Methods**: We retrospectively reviewed 174 consecutive perioperative spine patients referred to our institutional BHC between October 2019 and April 2024. Demographics, surgical characteristics, bone health status, laboratory and DXA results, pharmacologic management, contraindications, insurance-related barriers, and medication sequencing were collected. Surgical outcomes included hardware failure and revision surgery. Bone health response was assessed by follow-up DXA scans. **Results**: The cohort was predominantly female (78.2%) with a mean age of 71.9 years. Most patients were referred by neurosurgery (53.4%) or orthopedics (41.4%). Based on DXA and fragility fracture history, 27.0% had osteopenia, 56.3% osteoporosis, and 13.8% severe osteoporosis. Pharmacotherapy was prescribed in 146 patients (83.9%), most commonly romosozumab (32.9%), denosumab (22.6%), and abaloparatide (21.2%). Twenty-eight patients (16.1%) did not receive medication, primarily due to contraindications. Insurance-related barriers disproportionately affected anabolic agents, whereas denosumab had the lowest denial rate (15.2%, *p* = 0.0124). Sequential therapy was common (32.1%), most frequently romosozumab followed by denosumab. Hardware failure occurred in 11.5% of patients, with 5.7% requiring revision surgery. Among the 80 patients (46.0%) with follow-up DXA, 60% demonstrated improved bone mineral density, with an average T-score gain of 0.6 ± 0.5. **Conclusions**: Integration of a multidisciplinary BHC into perioperative spine care was feasible, associated with high rates of pharmacotherapy initiation, and demonstrated favorable early surgical outcomes compared to published complication rates in this population. Insurance and contraindications remain key barriers to anabolic therapy access, driving prescribing toward denosumab. Prospective studies are needed to confirm the impact of perioperative bone optimization on surgical durability, cost-effectiveness, and patient-centered outcomes.

## 1. Introduction

Osteoporosis, the most common metabolic bone disease, affects more than 50 million adults over age 50 in the United States and approximately 200 million worldwide [1,2,3]. With the aging population, its prevalence is projected to rise by about 33% by 2030 [1]. As osteoporosis becomes more common, so too will spine fractures and the number of osteoporotic patients who may be candidates for spine surgery, particularly spinal fusions. The prevalence of low bone density among spine surgery candidates is substantial: several reports indicate that over 30% have osteoporosis and more than 45% have osteopenia [4,5], and one study found that over 50% of women and nearly 14.5% of men older than 50 undergoing spine surgery met the criteria for osteoporosis [6].

Compromised bone density negatively impacts the clinical success of fixation-dependent procedures, such as fusions, and increases healthcare costs. Because both instrumentation and capacity to form a solid fusion relies on patient bone density, osteoporosis adversely affects outcomes [7] and is associated with greater difficulty with instrumentation and fixation, along with higher rates of postoperative complications, including pseudoarthrosis, spinal fracture, blood loss, and hardware failure [8,9,10,11,12,13,14]. An analysis using the National Inpatient Sample found that osteoporotic patients undergoing cervical spine surgery had a 1.5-times greater chance of requiring revision surgeries and 30% higher hospitalization costs [15]. In patients undergoing primary posterior thoracolumbar or lumbar fusion, pseudoarthrosis occurred in 46.2% of those with osteoporosis [8].

Despite evidence that poor bone health worsens spine surgery outcomes, routine screening and treatment for osteoporosis remain inconsistent among spine surgery patients [16], revealing a care gap and an opportunity for bone health optimization. Bone Health Clinics (BHCs) and Fracture Liaison Services (FLS) are designed to close this gap by improving identification, initiating evidence-based therapy, and follow-up care focused on osteoporosis management [17]. These programs have been shown to increase treatment rates following a fracture, reduce the rate of secondary fractures, and may lower healthcare costs [18,19,20,21,22,23].

The purpose of this study is to describe a multidisciplinary approach at a single academic institution using a newly created BHC housed within the orthopedic trauma department. This BHC represents a novel clinic started and run by advanced practice providers specifically focused on evaluating and optimizing the bone density of orthopedic and neurosurgical patients with fragility fractures or upcoming surgeries. We evaluated patient demographics, surgical characteristics, pharmacologic management, decision-making, and postoperative and clinical outcomes. We hypothesized that integrating a dedicated bone health service into perioperative spine care would be feasible, increase treatment initiation, and be associated with favorable early surgical outcomes.

## 2. Materials and Methods

### 2.1. Setting and Patient Population

We retrospectively reviewed 174 perioperative patients referred to the BHC between October 2019 and April 2024 for spine-related indications. Patients were advised for referral based on predefined internal Enhanced Recovery After Surgery (ERAS) guidelines that aim to identify high-risk spine fusion candidates whose bone health needed optimization [19,24]. These criteria, as detailed in Table 1, include clinical risk factors, any prior bone mineral density assessment, and multidisciplinary evaluations. Specific numeric thresholds for malnutrition were not uniformly imposed across all referring surgeons. Clinician documentation often reflected consideration of BMI, unintentional weight loss, and/or laboratory parameters (e.g., low albumin) [25,26]. Low CT Hounsfield units (HU) were coded when the evaluating surgeon and/or radiologist documented subthreshold trabecular HU on preoperative lumbar CT, excluding areas with focal degenerative sclerosis. While thresholds vary in practice, the literature supports HU < 110 as suggestive of osteoporosis [27,28]. A discussion of these criteria was facilitated by BHC staff with all attending spine surgeons at our institution. The referral workflow was multidisciplinary and typically initiated by the attending spine surgeon (Neurosurgery or Orthopedic Surgery) when a patient met these high-risk criteria. When placing a referral, the surgical team ordered a dual-energy x-ray absorptiometry (DXA) scan and labs as detailed in the data collection section. Following the surgeon’s referral trigger, the patient underwent standardized evaluation by a dedicated advanced practice provider (APP) within the BHC for comprehensive metabolic bone disease workup and optimization prior to or immediately following surgery.

Our perioperative cohort included patients referred to the BHC who had undergone spine fusion prior to their visit or those referred for optimization prior to a future spine surgery. Patients were included in this study if they had been formally evaluated through the BHC and received pre- and postoperative surgical follow-up. Referral patterns reflected the multidisciplinary integration of the service, with most patients referred from surgical specialties, particularly neurosurgery and orthopedics. The BHC protocol involves comprehensive screening, diagnosis, and initiation of pharmacotherapy. Patients who were identified as having complex or high-risk secondary causes of osteoporosis were referred to specialized services for co-management. This included direct referral to endocrinology services for individuals with suspected or confirmed diagnoses such as uncontrolled hyperparathyroidism, new or complicated Cushing’s syndrome, male hypogonadism requiring specialized therapy, or other complex metabolic or endocrine derangements that fall outside the scope of BHC management. Overall, this cohort represents a consecutive series of patients undergoing standardized evaluation and management at a single-institution bone health optimization program.

### 2.2. Bone Health Diagnosis

Bone health diagnoses were established in accordance with American Association of Clinical Endocrinologists (AACE) and the Bone Health and Osteoporosis Foundation (BHOF) guidelines [29,30]. Osteoporosis was defined as a T-score ≤ −2.5 at the lumbar spine, femoral neck, or total hip or the presence of a low-energy fragility fracture of the hip or spine regardless of bone mineral density (BMD). Osteopenia was defined as a T-score between −1.0 and −2.4. A diagnosis of osteoporosis was also given to patients in the osteopenia range who had a history of fragility fracture at any site, consistent with guideline-based practice. The lowest T-score across any site (lumbar spine, femoral neck, or total hip) was used for our primary analysis. This is due to local factors such as spine degenerative changes, presence of hardware, or prior fractures that may lead to falsely elevated BMD scores at the lumbar spine and mask systemic osteoporosis.

### 2.3. Data Collection

A retrospective review of the electronic medical record (Epic, Epic Systems, version May 2025) was performed for all eligible patients. Demographic variables collected included age, sex, body mass index (BMI), race and ethnicity, and health insurance status. Surgical variables included surgical indication, procedure and surgical approach, number of spinal levels fused, hardware failure (screw loosening, pullout, breakage, rod failure), and the length of surgical follow-up. Bone health assessments included DXA scans of the lumbar spine, femur, and distal radius. Laboratory studies obtained at or before the first BHC visit were also collected, including serum calcium, 25-hydroxy vitamin D, albumin, parathyroid hormone (PTH), C-terminal telopeptide (CTx), procollagen type 1 N-terminal propeptide (P1NP), and phosphorus. At their first BHC visit, patients completed a structured intake questionnaire documenting established risk factors for osteoporosis and fracture (Table 2). This included history of prior fragility fractures, family history of osteoporosis or hip fracture, history of falls, height loss, current or prior smoking history, alcohol and caffeine use, exercise habits, and dietary practices such as vegetarianism or veganism. The questionnaire also captured relevant medical comorbidities including rheumatoid arthritis, lupus, celiac disease, chronic kidney disease, seizure disorder, HIV/AIDS, gastric bypass, hepatitis B or C, COPD, Paget’s disease, hyperparathyroidism, diabetes, history of cancer, and/or long-term steroid use. Information on medication exposure was recorded for agents known to affect bone health, including bisphosphonates (alendronate, ibandronate, risedronate, zoledronate, pamidronate), PTH analogues (teriparatide, abaloparatide), romosozumab, denosumab, calcitonin, raloxifene, selective serotonin reuptake inhibitors (SSRIs), proton pump inhibitors (PPIs), oral glucocorticoids, anticonvulsants, opioids, anticoagulants, and hormone replacement therapy. Nutritional supplementation with calcium and vitamin D was also documented.

### 2.4. Surgical Outcomes and Diagnostic Criteria

Surgical outcomes, including rates of hardware failure (screw loosening, pullout, breakage, rod failure) and subsequent revision surgery, were retrospectively assessed via comprehensive electronic medical record review. The diagnosis of hardware failure was determined based on objective radiologic criteria seen on plain radiographs and/or CT scans.

Postoperative surveillance routinely included X-rays obtained at fixed time points, typically 6 weeks, 3 months, 6 months, and 12 months. If these X-rays revealed concerning findings (e.g., progressive lucency, hardware migration) or if the patients presented with clinical complaints (e.g., increasing pain or neurological symptoms), a CT scan was performed to further work up and confirm hardware failure. Specifically, screw loosening/pullout was defined as a lucency (halo) greater than 1 mm surrounding the screw threads or documented progressive screw migration on postoperative imaging. Screw or rod breakage and failure was confirmed by demonstrating complete hardware discontinuity on imaging. Interbody subsidence was defined as vertical migration or collapse of the interbody device into adjacent vertebral endplates, as measured radiographically or requiring surgical revision.

Treatment-related data included the first medication prescribed, sequencing of subsequent therapies, contraindications, patient preference, prior osteoporosis treatment, insurance-related barriers, and duration of therapy relative to surgery. All data were stored in REDCap (version 15.8.0) hosted at Stanford University. The study was approved by the Stanford University Institutional Review Board (Project 59326).

### 2.5. Statistical Analysis

Comparisons of categorical variables were conducted using chi-squared tests. Graphs were produced using version 10 of GraphPad Prism. Statistical significance was defined as *p* < 0.05, and all tests used were two-sided. All statistical and graphical analyses were conducted using version 4.3.2 of the R program language (R Foundation for Statistical Computing) and Microsoft Excel.

## 3. Results

### 3.1. Perioperative Patients

A total of 174 perioperative patients were referred to the BHC for evaluation and optimization of bone health for spine surgery (Table 3). The patients referred to the BHC represent a small subset of patients who received elective spine surgery during this time (<5%). Referral to BHC was at the discretion of the referring provider and was reserved for high-risk patients based on criteria in Table 1.

The cohort was predominantly female (78.2%) with a mean age of 71.9 ± 10.3 years. The average BMI was 27.6 ± 5.9. In terms of racial and ethnic distribution, most patients identified as White (66.7%), followed by Asian (15.5%), Hispanic or Latino (13.2%), Black or African American (1.1%), Native Hawaiian or Other Pacific Islander (0.6%), and American Indian or Alaska Native (0.6%). Referral sources reflected the multidisciplinary structure of the BHC (Table 4). The majority of the patients were referred by neurosurgery (53.4%) and by orthopedic surgery (41.4%), with smaller contributions from internal medicine (2.3%), family medicine (1.7%), and primary care (1.1%). The primary indications for surgery overwhelmingly involved degeneration (91.4% of patients), with the most frequent specific diagnoses being lumbar spinal stenosis (72.4%), spinal deformity (14.9%), and vertebral compression fractures (VCF) (14.4%).

In terms of surgical timing, 51.7% of patients began BHC treatment before a planned spine surgery, while 48.3% initiated treatment after undergoing spine surgery. The mean duration of bone health treatment prior to surgery was 255.3 ± 330.6 days, while treatment duration after surgery averaged 595.5 ± 494.1 days. The overall mean duration of BHC treatment was 586.7 ± 519.8 days, with a mean surgical follow-up time of 705.3 ± 570.4 days (Table 5). Surgical approaches were posterior (54.0%), combined anterior–posterior (31.0%), or anterior only (14.9%), with an average of 3.6 ± 2.8 levels fused (Table 6). Overall, single-location procedures (cervical, thoracic, lumbar) accounted for 49.5% of the cohort, while 50.5% involved multi-section interventions, which included fusions across contiguous regions (e.g., cervicothoracic, thoracolumbar, lumbosacral) or long fusions spanning three major sections (e.g., thoracic, lumbar, and sacral). The most common documented locations were the lumbar spine (25.3%) and the combined lumbosacral region (25.3%) (Table 6).

### 3.2. Bone Health Status and Risk Factors

In our study cohort, the mean lowest T-score across all sites was −2.3 ± 1.1 (Table 7). Based on DXA performed as part of BHC intake and fragility fracture history, 24 (13.8%) patients were diagnosed with severe osteoporosis, 98 patients (56.3%) with osteoporosis, and 47 (27.0%) with osteopenia. Only 5 patients (2.9%) had normal bone health. Prior fragility fractures were reported in 66 patients (67.3%) with osteoporosis and 17 patients (70.8%) with severe osteoporosis, while none were reported among patients with osteopenia or normal bone health. A total of 48 patients (25.9% of the cohort) were diagnosed with osteoporosis despite having a DXA T-score greater than −2.5 (mean T-score: −1.6 ± 1.1). This diagnosis was made based on the presence of a fragility fracture, which is clinically sufficient for diagnosing osteoporosis regardless of the bone mineral density score. Of these 48 patients, 45 (93.8%) had a documented fragility fracture.

### 3.3. Patient-Reported Risk Factors (Intake Questionnaire)

The cohort demonstrated a significantly elevated risk profile based on history alone (Table 8). Almost half of all patients had a prior fragility fracture (47.7%), and low energy vertebral compression fractures (VCFs) were reported in 31.6% of patients. Furthermore, 24.1% reported a history of more than two falls in the preceding year. In terms of comorbidities and medication exposure known to negatively impact bone health, prominent findings included: long-term proton pump inhibitor (PPI) use (30.5%), which interferes with calcium absorption; hypothyroidism (23.6%) and diabetes (16.7%), both conditions associated with decreased bone quality; and long-term steroid use (10.9%) or prior cancer/radiation therapy (21.3%). Only 29.9% of the patients were current or former smokers, and a majority displayed limited or no regular weight-bearing exercise (58.1%). These non-densitometric factors strongly validated the decision to enroll these patients in the BHC for optimization.

### 3.4. Pharmacologic Management

**Figure 1:**
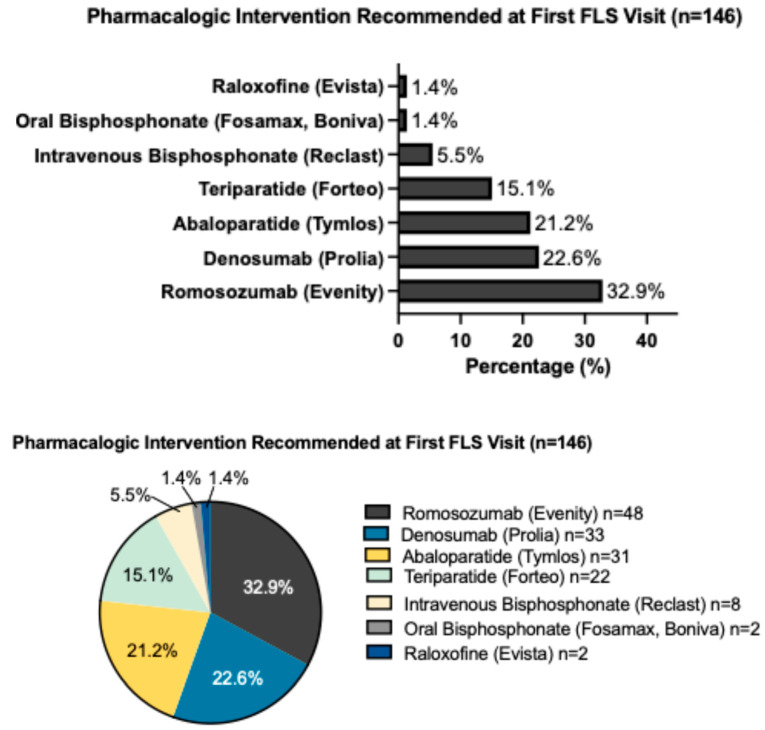
Pharmacologic intervention recommended at first FLS visit.

Of the 174 patients referred for perioperative bone health optimization, 146 (83.9%) were prescribed pharmacotherapy. The most commonly prescribed first-line agent was romosozumab (Evenity), initiated in 48 patients (32.9%). The second most commonly used agent was denosumab (Prolia) in 33 patients (22.6%), abaloparatide (Tymlos) in 31 patients (21.2%), and teriparatide (Forteo) in 22 patients (15.1%). Smaller numbers of patients were started on intravenous bisphosphonates (Reclast, *n* = 8, 5.5%), oral bisphosphonates (alendronate/ibandronate, *n* = 2, 1.4%), or raloxifene (Evista, *n* = 2, 1.4%) (Table 9, Figure 1). Further analysis of therapeutic choice (Table 10) revealed that romosozumab was the most frequently selected first-line therapy for patients diagnosed with both osteoporosis (36.4%) and severe osteoporosis (45.8%), consistent with our anabolic-first treatment strategy for the highest-risk surgical candidates. Twenty-eight patients (16.1%) did not receive pharmacologic therapy (Table 11). Most of these patients had osteopenia (*n* = 13) or normal bone health (*n* = 5), where clinicians deemed pharmacotherapy inappropriate or patients opted to not start medications. Contraindications accounted for four of these cases.

**Figure 2:**
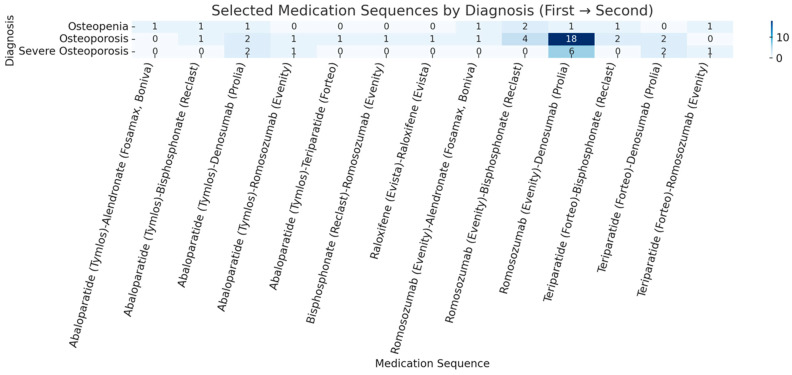
Heatmap of number of patients in each medication sequence by diagnosis.

Medication sequencing was common, with 56 patients undergoing at least two pharmacologic regimens (Table 12, Figure 2). The most frequent sequence was romosozumab followed by denosumab (*n* = 25), reflecting an anabolic-to-antiresorptive strategy. Other frequent sequences included romosozumab followed by intravenous bisphosphonate (*n* = 6), abaloparatide followed by denosumab (*n* = 5), and teriparatide followed by denosumab (*n* = 4). Less common sequences included bisphosphonate-to-romosozumab and raloxifene-to-raloxifene transitions, which reflected patient-specific contraindications or insurance-driven constraints rather than first-line clinical preference.

Duration analysis demonstrated that most anabolic therapies were maintained for approximately 9–24 months before transition, aligning with guideline recommendations for limited anabolic use [31]. In our cohort, for example, romosozumab was administered for a mean of 298.8 days, followed by denosumab with a short transition gap (mean 66.6 days). In contrast, some transitions reflected prolonged anabolic use: teriparatide averaged 619.8 days before denosumab initiation, and certain bisphosphonate-to-romosozumab sequences persisted beyond 2.5 years (mean 1019 days before transition) (Table 13). Importantly, all bisphosphonate-to-romosozumab sequences reflected preexisting bisphosphonate therapy initiated by outside providers prior to referral; our BHC did not start bisphosphonates and then switch to an anabolic for perioperative optimization. When clinically appropriate, we transitioned patients from preexisting bisphosphates to anabolic therapy, consistent with our anabolic-first approach. These patterns highlight both clinical tailoring and insurance approval timelines as real-world drivers of treatment sequencing.

### 3.5. Factors Influencing Medication Selection

The choice of pharmacologic therapy was influenced by multiple factors, most notably contraindications, insurance coverage, and patient preference (Table 14). Contraindications were the leading determinant, impacting 27% of patients. These were most frequently related to PTH analogues (19.5%; high beam radiation therapy history, renal impairment, and/or hypercalcemia) and oral bisphosphonates (11.5%; history of gastroesophageal reflux disease), with smaller proportions of contraindications for romosozumab (3.4%; history of stroke or myocardial infarction and/or hypocalcemia), intravenous bisphosphonates (1.7%; renal impairment), and denosumab (0.6%; hypocalcemia). A subset of patients (7.5%) had multiple contraindications that further narrowed therapeutic options. Insurance-related barriers hindered medication access for 43 patients (24.7% of the cohort). Government/public payors accounted for the largest category of access issues, affecting 33 patients (19.0%). Private payors accounted for barriers in 8 patients (4.6%). Notably, 1.1% of patients had no active insurance at the time of evaluation (Table 15).

To further understand the role of insurance in treatment selection, we examined the distribution of insurance issues by medication type (Table 16). This analysis highlights the percentage of patients who experienced insurance-related barriers versus those without such barriers for each prescribed agent. Nearly half of patients prescribed abaloparatide (48.4%) and teriparatide (42.9%) encountered insurance-related issues, as did 50% of those prescribed intravenous or oral bisphosphonates. In contrast, denosumab was associated with significantly fewer insurance problems, with only 15.2% of patients experiencing coverage issues compared with 84.8% without (*p* = 0.0124). Romosozumab showed intermediate levels of insurance-related barriers, affecting 35.4% of patients.

### 3.6. Surgical Outcomes and Bone Health Response

At a mean postoperative follow-up of 2.9 ± 1.7 years, 20 patients (11.5%) experienced hardware failure (Table 17). To ensure comprehensive preoperative evaluation and postoperative surveillance for hardware complications, patients frequently received multiple imaging modalities. The initial workup for the cohort included a preoperative X-ray for 117 patients, a preoperative CT scan for 75 patients, and a preoperative MRI for 53 patients. Following treatment and surgery, postoperative surveillance consisted of a postoperative X-ray for 154 patients, a postoperative MRI for 65 patients, and a postoperative CT scan for 59 patients. The most frequent complication was screw loosening (6.9%), followed by screw breakage (2.3%), rod breakage (2.3%), screw pullout (1.7%), and interbody subsidence (0.6%). Hardware failure occurred across diagnostic subgroups, with the highest absolute number among patients with osteoporosis (*n* = 11, 11.2% of that subgroup), followed by severe osteoporosis (*n* = 3, 12.5%) and osteopenia (*n* = 5, 10.6%). Notably, only one patient with normal bone density experienced hardware failure, though the small group size limits interpretation, and exact bone quality is difficult to determine.

Revision surgery was required in 10 patients (5.7%), reflecting half of those with hardware failure. Bone health outcomes were also favorable among patients with longitudinal follow-up. Of the 80 patients (46.0%) who underwent a repeat DXA scan following initiation of therapy, 48 (60.0%) demonstrated improvement in their lowest T-score, with an average gain of 0.6 ± 0.5. Mean T-scores improved from −2.5 ± 1.0 at baseline to −2.2 ± 1.2 at follow-up. These improvements were particularly notable in patients treated with an anabolic-to-antiresorptive sequence, reinforcing the clinical rationale for sequential therapy (Table 12 and Figure 2). This is in line with the 2024 American Society for Bone and Mineral Research position statement supporting this treatment sequence [32].

## 4. Discussion

Our institution developed a Bone Health Clinic housed in the orthopedic trauma department in 2019 for fragility fracture and metabolic bone disease, focused on identifying risks, investigating causes, and implementing treatment plans to reduce the risk of future fractures and optimize surgical outcomes. The clinical philosophy driving the BHC emphasizes the long-term management of osteoporosis as a chronic systemic disease. The clinic operates at the intersection of neurosurgery, orthopedic surgery, endocrinology, and rheumatology. In this study, we describe our approach to perioperative bone optimization for spinal fusions. Spine surgery (and spinal fusion in particular) is challenging in the elderly due to a high prevalence of poor bone quality [33,34,35]. Osteoporotic patients undergoing spinal fusion face increased perioperative complications, including vertebral fractures after instrumentation, pseudoarthrosis, estimated blood loss, and hardware failures [9,36,37,38,39,40,41]. They are also more likely to require revision surgery, have longer hospitalizations, and incur higher inpatient and outpatient costs compared to non-osteoporotic patients. Given these risks, perioperative bone optimization may be beneficial for successful outcomes [15,42,43].

More than 70% of patients referred to the clinic for spine surgery bone optimization received an osteoporosis diagnosis, with an additional 47% diagnosed with osteopenia, underscoring the high prevalence of poor bone quality in this population. Because the average T-score among those diagnosed with osteoporosis was −2.3, we examined patients with an osteoporosis diagnosis despite osteopenia-range DXA scores. Nearly 94% of this subset had prior fragility fractures. Consistent with diagnostic criteria, a documented low-energy fracture (e.g., hip or vertebral) supersedes screening tools such as DXA and should prompt treatment and perioperative optimization. Accordingly, fracture history was prioritized over densitometric thresholds when making therapeutic decisions in this cohort. The remainder were diagnosed by another provider prior to starting treatment at the BHC or due to poor bone quality found intraoperatively. Thus, a substantial proportion of patients carry an osteoporosis diagnosis based on fragility fractures rather than densitometric criteria alone, a critical consideration for treatment decisions. Notably, a previous study found that only 25.6% of patients with primary fragility fractures had received a DXA scan or initiated pharmacotherapy in the two years before the fracture [44].

Nearly 52% of patients started treatment at the BHC preoperatively, with a mean lead time of 8 months prior to surgery, consistent with current clinical practice [45,46]. Although surgery was delayed for these patients, they were generally motivated to optimize their bone health and expressed appreciation for referral. Following comprehensive evaluation, patients with evidence of poor bone health were commonly prescribed medications. Overall, 84% received first-line pharmacotherapy, guided by initial labs, screening for comorbidities and contraindications, and the 2020 AACE Clinical Practice Guidelines for the diagnosis and treatment of postmenopausal osteoporosis [30]. This approach targeted patients for drug interventions who were classified as “high-risk” based on clinical factors beyond BMD alone, including those with a recent or multiple fragility fractures (within the last 12 months), fracture occurrence while on approved osteoporosis therapy or while taking long-term corticosteroids, a low T-score (≤−3.0) at the spine, femoral neck, or total hip, a high risk for falls, or a very high FRAX^®^ probability (>30% for major osteoporotic fracture or >4.5% for hip fracture).

The AACE guidelines recommend anabolic agents first: these include teriparatide, ababaloparatide, or romosozumab. This is why our BHC protocol involves an anabolic-first approach, aimed at sequential therapies to promote rapid and robust bone quality improvement in this high-risk surgical population; 77% of those treated were placed on anabolic agents (romosozumab, abaloparatide, and teriparatide). If the first-line anabolic agents were contraindicated or if patients were unable to obtain insurance coverage, antiresorptive agents, such as denosumab, were considered as a secondary option based off results from this 2016 study [47]. This approach, while distinct from traditional osteoporosis treatment pathways, reflects the current standard of care for our high-risk patient population [40]. Patients undergoing spine fusion surgery, particularly those with existing osteoporosis or recent fragility fractures, are considered to be at high risk, necessitating rapid and robust bone quality improvement [48]. Anabolic agents are increasingly favored perioperatively because they promote bone formation, improve bone mass and architecture, and reduce fracture risk more than traditional antiresorptives [49,50,51].

Teriparatide consistently reduces vertebral fracture risk and increases spine BMD in randomized trials [52], and human studies suggest it enhances fusion and lowers pedicle screw loosening rates [52,53]. Several studies indicate it may be superior to bisphosphonates preoperatively [53,54,55], aligning with clinical practice. In a survey of 18 multidisciplinary physicians, most recommended anabolic agents such as teriparatide or abaloparatide as first-line therapy prior to elective spine reconstruction [45]. While abaloparatide improves BMD, bone architecture, and fracture protection, it has not yet been studied in spine surgery outcomes [49].

Romosozumab acts as both an anabolic and antiresorptive agent by binding to and inhibiting sclerostin, a negative regulator of osteoblast activity [56]. It has shown significant reductions in vertebral fracture risk and gains in bone mass and strength at the lumbar spine, though its effectiveness specific to spine surgery requires further study [57,58,59]. Emerging evidence suggests administration two months before surgery can enhance BMD and bone strength, improving surgical outcomes and reducing complications in corrective spinal fusion surgery [60]. In our BHC, romosozumab was the most frequently used medication among perioperative patients (33%), which may reflect both clinical efficacy and patient preference. Notably, several studies found suboptimal adherence to teriparatide [61,62,63]. Adherence may be higher with monthly romosozumab injections compared with daily teriparatide.

We also examined factors influencing treatment choice. Contraindications were the primary determinant of the agent prescribed, while route of administration, dosing interval, and travel distance substantially influenced patient preference. Insurance coverage and co-payment frequently dictated final selection. Most insurance-related adjustments required opting for alternative agents in place of teriparatide or abaloparatide because of high co-payments or insurance denials despite appeals. Denosumab was commonly prescribed and had low denial rates, which likely contributed to it being the second most frequently used medication despite its antiresorptive mechanism. An anabolic-to-antiresorptive sequence was completed by 48 (27.6%) patients, consistent with literature showing that initiating therapy with an anabolic agent followed by an antiresorptive yields greater gains in bone mass, whereas starting with a bisphosphonate may blunt subsequent anabolic efficacy [64,65]. It should also be noted that failure to maintain bone health by following anabolic treatment with an antiresorptive medication results in rapid loss of the gains achieved in bone quality [64,66].

With mean post-surgical follow-up approaching two years, our overall hardware failure rate was 11.5%, with failure types including screw loosening, pullout, and rod breaking. The most common failure was screw loosening (6.9%). Notably, we observed rod breakage in 4 cases (2.3%). While the exact mechanism of failure for the rods was not uniformly documented, rod breakage in spinal fusion constructs is widely recognized in the literature as a strong surrogate indicator of pseudoarthrosis (non-union). This failure occurs due to sustained, cyclical fatigue loading on the implant caused by a failed bony fusion. These 4 cases likely represent patients with underlying non-union, a complication exacerbated in patients with poor bone quality [40].

Only 10 patients (5.7%) required revision surgery for hardware-related complications. Prior studies report substantially higher complication burdens in osteoporotic spine populations, including overall prevalence rates near 32.1%, early complications (e.g., pedicle and compression fractures) in 13%, pseudoarthrosis with instrumentation failure in 11%, loosening of instrumentation in 7% [8,9]. Another study found implant loosening of up to 48.6% with revision rates of 26% in elderly patients with poor bone health [10]. While definitions and follow-up intervals vary across studies, our observed rates appear lower than many published cohorts. The average follow-up period for our spine surgery cohort was 2.9 ± 1.7 years (705.3 ± 570.4 days), exceeding the established benchmark of 12 to 24 months commonly employed for evaluating radiographic fusion outcomes and hardware-related complications, such as screw loosening and pullout, in the spine surgery population [67,68,69,70]. This robust duration facilitates accurate assessment of both fusion success, typically defined by radiographic criteria on CT or X-ray, and the majority of early-to-mid-term device-related failures [67,68,69,70]. Notably, clinical trials and retrospective studies analyzing the impact of antifracture medications (e.g., teriparatide, romosozumab) on fusion rates and implant integrity in high-risk populations routinely employ follow-up intervals within this range, further validating our dataset’s ability to capture relevant clinical events, including an observed 11.5% hardware failure rate and the efficacy of our treatment protocols [63,64,65]. Systematic screening and treatment within a dedicated BHC, frequent use of anabolic therapy with adequate preoperative lead time, multidisciplinary coordination that influences surgical planning, and postoperative follow-up may contribute to these differences. Causal effect cannot be established in this small retrospective series and must be interpreted cautiously given potential confounding by patient selection, differing surgical technique by provider, and differences in patient comorbidity profiles.

Of the 80 patients with follow-up DXA scans, 60% had improved T-scores on their most recent study, supporting the effectiveness of BHC treatment. While improved densitometry plausibly contributes to reduced hardware complications and revision surgery, prospective data are needed to confirm the relationship between perioperative bone optimization, changes in BMD, and surgical outcomes.

### Limitations

This single-institution study lacks a control group, limiting causal inference and generalizability. Additionally, it is difficult to compare differences in surgical outcomes. The retrospective design introduces potential selection bias (e.g., patients willing to delay surgery and engage in optimization may differ from those who proceed without optimization). Follow-up duration was variable, and loss to follow-up may underestimate late complications or overestimate treatment success. Longer term follow-up (5+ years) is needed to assess for long-term hardware failure. In addition, we acknowledge that using either x-ray or CT scan to detect hardware failure may lead to under-detection of some types of hardware failure, such as subtle screw loosening. However, given the significant amount of increased radiation with CT scans, this is not standard of care for patients who are doing well postoperatively at our institution. CT scans were therefore only obtained for patients in whom hardware failure was suspected due to x-rays or clinical concerns. Another limitation is that due to the relatively low hardware failure rate and our sample size, we are not adequately powered to detect differences between specific medication prescriptions across patients with different BMD classifications. However, our results do suggest that our anabolic-first strategy has promising results for high-risk patients. Finally, therapy management were influenced by insurance coverage and co-payments, which vary across regions and health systems. Surgical techniques were not standardized and may confound outcome comparisons.

## 5. Conclusions

Spine surgeons should be attuned to the complex issues surrounding bone health and perioperative bone optimization, including medication options, indications, contraindications, patient preferences, and insurance constraints. Our multidisciplinary BHC model demonstrates that systematic screening and targeted pharmacotherapy, often with an anabolic-first approach, are feasible in routine practice, accepted by patients, and associated with improved BMD and lower hardware complication rates relative to many published cohorts. We recommend integrating multidisciplinary bone health services into spine care pathways to identify at-risk patients early, initiate timely optimization, and coordinate perioperative management. Future work should include prospective, controlled studies to define the optimal timing, duration, and sequencing of anabolic and antiresorptive therapies; evaluate patient-centered outcomes and cost-effectiveness; and develop standardized protocols for screening, treatment, and longitudinal follow-up. Broader collaborations across orthopedic surgery, neurosurgery, endocrinology, rheumatology, and primary care will be essential to reduce fracture risk and improve surgical outcomes for patients with compromised bone health.

## Figures and Tables

**Table 1:** Criteria for advised referral to bone health optimization in high-risk spine fusion candidates.

Criterion	Description
Age	Age ≥ 50, advanced age, or postmenopausal status
Comorbidities	Chronic steroid use, diabetes, autoimmune disorders, renal disease
Preoperative Osteoporosis Risk	History of osteoporosis, osteopenia, prior fragility fractures
Nutritional Status	Malnutrition *, low BMI, vitamin D deficiency
Bone Health Assessment	DXA scan (T-score ≤ −1.0), CT Hounsfield units low *, abnormal labs
Fracture Risk Tools	FRAX score in osteoporotic or high-risk range
Multidisciplinary Concern	Clinical concern from surgeon or perioperative geriatrics team

* Malnutrition and low CT HU were not defined by a single study-wide numeric threshold. See Methods for details and literature-based thresholds provided for context.

**Table 2:** Bone clinic patient intake questionnaire.

Who referred you to this program?	
2. Current or recent fracture?	
3. Have you sustained any other bone fractures since you turned 50?	
4. Have you had more than two falls in the past year?	
5. Have you had a Bone Density Scan or DXA in the past two years?	
6. How active are you and what is your exercise of choice?	
7. Have you had height loss or feel shorter since your 20s?	
8. If female, are you still having menstrual periods?	
9. If female, have you had a hysterectomy?	
10. If male, have you ever been told you have low testosterone?	
11. Have you ever taken hormone replacement therapy?	
12. Did either of your parents have a hip fracture after the age of 50 or any family member have osteoporosis?	
13. Are you vegetarian or vegan?	
14. Do you currently smoke, or did you?	
15. Do you drink alcohol?	
16. How many caffeinated beverages do you have a day (1 serving = 8 oz.)?	
17. Have you ever been diagnosed or treated for the following? (check all that apply)	
⎕ Rheumatoid Arthritis	⎕ Kidney stones
⎕ Lupus	⎕ Seizure disorder
⎕ Celiac disease or irritable bowel	⎕ HIV/AIDS
⎕ Gastric Bypass	⎕ Hepatitis B or C
⎕ COPD	⎕ Paget’s Disease
⎕ GERD or Acid Reflux	⎕ Prostate Cancer
⎕ Hyperparathyroidism	⎕ Breast Cancer
⎕ Hypothyroidism	⎕ Long-term steroid use
⎕ Diabetes	
18. Are you currently or have you taken any of the following medications? (If so, for how long?)	
⎕ Fosamax (Alendronate)	⎕ Anticonvulsants (Gabapentin, Lyrical, Lamictal)
⎕ Didronel (Etidronate)	⎕ Anticoagulants (Heparin, Warfarin)
⎕ Boniva (Ibandronate)	⎕ Opioids (Oxycodone/Oxycontin)
⎕ Aredia (Pamidronate)	⎕ Oral steroids (prednisone)
⎕ Actonel (Risedronate)	⎕ PPI’s (Omeprazole, Prilosec, Nexium)
⎕ Reclast (Zoledronate)	⎕ SSRI’s (Lexapro, Celexa, Sertraline)
⎕ Fortical (Calcitonin)	
⎕ Miacalcin (nasal spray)	
⎕ Evista (Raloxifene)	
⎕ Forteo (Teriparatide)	
⎕ Prolia (Denosumab)	
19. Are you currently taking any nutritional supplements?	
Calcium?	
Vitamin D?	
20. Have you ever been treated for cancer with high beam radiation or had radioactive implants?	
21. Have you suffered a stroke or heart attack in the past 12 months?	

**Table 3:** Baseline demographics and characteristics of cohort.

Characteristic	*n* = 174
Age (years), mean ± SD	71.9 ± 10.3
Sex, *n* (%)	
Female	136 (78.2)
Male	38 (21.8)
BMI, mean ± SD	27.6 ± 5.9
Race/Ethnicity, mean ± SD	
American Indian or Alaskan Native	1 (0.6)
Asian	27 (15.5)
Black of African American	2 (1.1)
Hispanic or Latino	23 (13.2)
Native Hawaiian or Other Pacific Islander	1 (0.6)
White	116 (66.7)
Other	10 (5.7)

**Table 4:** Referral department, surgical indication, and surgical timing.

Category	*n* = 174
**Referral Department *n* (%)**	
Neurosurgery	93 (53.4)
Orthopedics	72 (41.4)
Internal Medicine	4 (2.3)
Family Medicine	3 (1.7)
Primary Care	2 (1.1)
Indication for Surgery * *n* (%)	
Degeneration	159 (91.4)
Trauma	13 (7.5)
Tumor	5 (2.9)
Infection	2 (1.1)
Other	2 (1.1)
Diagnosis * *n* (%)	
Lumbar Spinal Stenosis	126 (72.4)
Spinal Deformity (Scoliosis/Kyphosis/Kyphoscoliosis)	26 (14.9)
Vertebral Compression Fracture (VCF)	25 (14.4)
Degenerative Spondylolisthesis	13 (7.5)
**Timing of Surgery, *n* (%)**	
Began FLS Treatment After Surgery	84 (48.3)
Began FLS Treatment Before Surgery	90 (51.7)

* Some patients had more than one surgical indication documented.

**Table 5:** Timing and duration of bone health treatment relative to spine surgery.

Timing of Treatment, Mean ± SD	Treatment Length (Days)
Treatment Duration Before Surgery	255.3 ± 330.6
Treatment Duration After Surgery	595.5 ± 494.1
Overall Duration of Treatment at FLS	586.7 ± 519.8
Days of Surgical Follow-Up	705.3 ± 570.4

**Table 6:** Surgical characteristics.

**Surgical Approach, *n* (%)**	
Anterior	26 (14.9)
Posterior	94 (54.0)
Combined	54 (31.0)
Spinal Location, *n* (%)	
Cervical	33 (19.0)
Cervicothoracic	9 (5.2)
Cervicothoracic-lumbar	1 (0.6)
Cervical, Thoracolumbar-pelvis	1 (0.6)
Thoracic	9 (5.2)
Thoracolumbar	12 (6.9)
Thoracolumbar-pelvis	1 (0.6)
Thoraco-lumbosacral	18 (10.3)
Lumbar	44 (25.3)
Lumbosacral	44 (25.3)
Sacroiliac	2 (1.1)
Surgical Procedure, *n* (%)	
Anterior/Lateral Interbody Fusion (ALIF, XLIF, LLIF)	55 (31.6)
Posterior Interbody Fusion (TLIF, PLIF)	24 (13.8)
PSF Only (Posterior Spinal Fusion without Interbody)	64 (36.8)
Cervical Fusion (ACDF, PCDF)	31 (17.8)
**Number of Levels Fused, mean ± SD**	3.6 ± 2.8

**Table 7:** Bone health diagnosis and prevalence of fragility fractures.

Diagnosis	Count, *n* (%)	Fragility Fracture (% per Row)	Lowest DXA Scan T-Score, Mean ± SD
Normal Bone Health	5 (2.9)	0	−0.5 ± 0.4
Osteopenia	47 (27.0)	0	−1.8 ± 0.5
Osteoporosis	98 (56.3)	66 (67.3)	−2.3 ± 1.1
Severe Osteoporosis	24 (13.8)	17 (70.8)	−3.8 ± 0.9
Total	174		−2.3 ± 1.1
Osteoporosis w/DXA > −2.5	48 (25.9)	45 (93.8)	−1.6 ± 1.1

**Table 8:** Bone clinic intake questionnaire responses.

Risk Factor	*n* (%)
History of more than two falls in past year	42 (24.1)
History of low energy vertebral compression fracture	55 (31.6)
Height loss since 20s	107 (61.5)
History of non-spine failed implants, periprosthetic lucency	18 (10.3)
Prior fragility fracture	83 (47.7)
If female, menopause at age < 45	50 (28.7)
If male, history of low testosterone	12 (6.9)
History of hormone replacement therapy	
Current	14 (8.0)
Former	40 (23.0)
Never	120 (69.0)
Parents with hip fracture after age of 50 or family history of osteoporosis	67 (38.5)
Vegetarian	8 (4.6)
Vegan	2 (1.1)
Exercise	
Active	42 (24.1)
Moderate	31 (17.8)
Limited	68 (39.1)
None	33 (19.0)
History of tobacco use	
Current	12 (6.9)
Former	40 (23.0)
Never	122 (70.1)
History of alcohol use	
Current	63 (36.2)
Former	10 (5.7)
Never	101 (58.0)
Caffeine Intake	
No caffeinated beverages	38 (21.8)
Less than 3 servings a day	123 (70.7)
More than 3 servings a day	13 (7.5)
Calcium supplementation	98 56.3)
Vitamin D supplementation	122 (70.1)
History of cancer	37 (21.3)
History of cancer treatment with high beam radiation	19 (10.9)
History of stroke or heart attack in the past 12 months	4 (2.3)
History of renal disease	22 (12.6)
Other Diagnoses	
Rheumatoid Arthritis	17 (9.8)
Lupus	1 (0.7)
Celiac disease or irritable bowel	3 (1.7)
Gastric bypass	2 (1.1)
COPD	10 (5.7)
GERD or acid reflux	76 (4.4)
Hyperparathyroidism	7 (4.0)
Hypothyroidism	41 (23.6)
Diabetes	29 (16.7)
Kidney stones	18 (10.3)
Seizure disorder	2 (1.1)
HIV/AIDS	2 (1.1)
Hepatitis B or C	6 (3.4)
Paget’s Disease	0 (0)
Prostate Cancer	6 (3.4)
Breast Cancer	8 (4.6)
Long-term steroid use	19 (10.9)
Medications	
Fosamax (Alendronate)	36 (20.7)
Didronel (Etidronate)	1 (0.6)
Boniva (Ibandronate)	10 (5.7)
Acredia (Pamidronate)	0 (0)
Actonel (Risedronate)	1 (0.6)
Reclast (Zoledronate)	3 (1.7)
Fortical (Calcitonin)	2 (1.1)
Miacalcin (nasal spray)	2 (1.1)
Evista (Raloxifene)	2 (1.1)
Forteo (Teriparatide)	7 (4.0)
Prolia (Denosumab)	10 (5.7)
Anticonvulsants (Gabapentin, Lyrical, Lamictal)	16 (9.2)
Anticoagulants (Heparin, Warfarin)	1 (0.6)
Opioids (Oxycodone/Oxycontin)	8 (4.6)
Oral steroids (prednisone)	21 (12.1)
PPI’s (Omeprazole, Prilosec, Nexium)	53 (30.5)
SSRI’s (Lexapro, Celexa, Sertraline)	21 (12.1)

**Table 9:** First medication prescribed at FLS visit.

First Medication Prescribed at FLS Visit	*n* = 146	Mean Lowest T-Score ± SD
Romosozumab (Evenity)	48	−2.8 ± 1.0
Denosumab (Prolia)	33	−2.3 ± 1.0
Abaloparatide (Tymlos)	31	−2.1 ± 0.9
Teriparatide (Forteo)	22	−2.9 ± 0.9
Intravenous Bisphosphonate (Reclast)	8	−2.8 ± 0.7
Oral Bisphosphonate (Fosamax, Boniva)	2	−2.2 ± 0.4
Raloxofine (Evista)	2	−2.5 ± 1.3

**Table 10:** First-line pharmacologic agent stratified by bone health diagnosis.

Medication	Osteopenia *n* (%)	Osteoporosis *n* (%)	Severe Osteoporosis *n* (%)	*p* = 0.19 (χ^2^ Test)
Romosozumab (Evenity)	5 (14.7)	32 (36.4)	11 (45.8)	
Denosumab (Prolia)	11 (32.4)	21 (23.9)	1 (4.2)	
Abaloparatide (Tymlos)	9 (26.5)	18 (20.5)	4 (16.7)	
Teriparatide (Forteo)	5 (14.7)	12 (13.6)	5 (20.8)	
Intravenous Bisphosphonate (Reclast)	3 (8.8)	3 (3.4)	2 (8.3)	
Oral Bisphosphonate (Fosamax, Boniva)	0 (0)	1 (1.1)	1 (4.2)	
Raloxofine (Evista)	1 (2.9)	1 (1.1)	0 (0)	
Total	34	88	24	

**Table 11:** Patients without pharmacologic management (*n* = 28).

FLS Diagnosis	*n* = 28	Contraindication
Osteopenia	13	2
Osteoporosis	10	0
Normal bone health	5	2

**Table 12:** Medication sequences and timelines for all patients with two medications.

First Medication	Second Medication	Count
Romosozumab (Evenity)	Denosumab (Prolia)	25
Romosozumab (Evenity)	Intravenous Bisphosphonate (Reclast)	6
Abaloparatide (Tymlos)	Denosumab (Prolia)	5
Teriparatide (Forteo)	Denosumab (Prolia)	4
Teriparatide (Forteo)	Intravenous Bisphosphonate (Reclast)	3
Abaloparatide (Tymlos)	Intravenous Bisphosphonate (Reclast)	2
Abaloparatide (Tymlos)	Romosozumab (Evenity)	2
Romosozumab (Evenity)	Oral Bisphosphonate (Fosamax, Boniva)	2
Teriparatide (Forteo)	Romosozumab (Evenity)	2
Abaloparatide (Tymlos)	Oral Bisphosphonate (Fosamax, Boniva)	1
Abaloparatide (Tymlos)	Teriparatide (Forteo)	1
Bisphosphonate (Reclast)	Romosozumab (Evenity)	1
Denosumab (Prolia)	Abaloparatide (Tymlos)	1
Raloxifene (Evista)	Raloxifene (Evista)	1
	Total	56

**Table 13:** Medication duration for all patients with two medications.

First Medication	Second Medication	*n* = 54 *	Duration (Days) of FIRST Medication, Median [IQR], Mean ± SD	Gap (Days) Between First and Second Medication
Romosozumab (Evenity)	Denosumab (Prolia)	25	331 [IQR 59], mean 298.8 ± 147.3	31 [IQR 28], mean 66.6 ± 216.8
Romosozumab (Evenity)	Bisphosphonate (Reclast)	6	348 [IQR 26], mean 350.3 ± 19.7	21 [IQR 9], mean 18.2 ± 13.1
Abaloparatide (Tymlos)	Denosumab (Prolia)	4	274 [IQR 185], mean 275.8 ± 107.7	9 [IQR 105], mean 105.5 ± 198.4
Teriparatide (Forteo)	Denosumab (Prolia)	4	733 [IQR 301], mean 619.8 ± 388.9	8 [IQR 58], mean 2.5 ± 74.5
Teriparatide (Forteo)	Bisphosphonate (Reclast)	3	638 [IQR 144], mean 666.7 ± 146.1	34 [IQR 34], mean 34.0 ± 48.1
Abaloparatide (Tymlos)	Bisphosphonate (Reclast)	2	548 [IQR 182], mean 548.5 ± 256.7	20 [IQR 6], mean 20.5 ± 7.8
Abaloparatide (Tymlos)	Romosozumab (Evenity)	2	20 [IQR 8], mean 20.5 ± 10.6	46 [IQR 38], mean 46.5 ± 54.4
Romosozumab (Evenity)	Alendronate (Fosamax, Boniva)	2	202 [IQR 116], mean 202.0 ± 164.0	482 [IQR 0], mean 482.0 ± nan
Teriparatide (Forteo)	Romosozumab (Evenity)	2	411 [IQR 402], mean 411.0 ± 568.5	96 [IQR 96], mean 95.5 ± 135.1
Abaloparatide (Tymlos)	Alendronate (Fosamax, Boniva)	1	230 [IQR 0], mean 230.0 ± nan	73 [IQR 0], mean 73.0 ± nan
Abaloparatide (Tymlos)	Teriparatide (Forteo)	1	365 [IQR 0], mean 365.0 ± nan	1163 [IQR 0], mean 1163.0 ± nan
Bisphosphonate (Reclast)	Romosozumab (Evenity)	1	1019 [IQR 0], mean 1019.0 ± nan	24 [IQR 0], mean 24.0 ± nan
Raloxifene (Evista)	Raloxifene (Evista)	1	365 [IQR 0], mean 365.0 ± nan	135 [IQR 0], mean 135.0 ± nan

* Timing/dates not available for *n* = 2 patients after chart review.

**Table 14:** Factors influencing medication selection.

Factors Influencing Medication Decision	*n*, %
Contraindications *	47 (27%)
Oral Bisphosphonate	20 (11.5)
PTH Analogues	34 (19.5)
Romosozumab (Evenity)	6 (3.4)
Intravenous Bisphosphonate (Reclast)	3 (1.7)
Denosumab (Prolia)	1 (0.6)
Patients with Multiple Contraindications	13 (7.5)
Patient Preference	23 (13.2)
Previous Medication Failure	8 (4.6)
Insurance	43 (24.7)

* Some patients had more than one contraindication. Percentages are out of the total number of patients.

**Table 15:** Insurance-related barriers to osteoporosis medication access.

Insurance Category	*n*, % of Total Cohort
Government/Public Payors	33 (19.0)
Private Payors	8 (4.6)
No Active Insurance/Other	2 (1.1)
Total Insurance-Related Barriers	43 (24.7)

**Table 16:** Insurance-related barriers stratified by osteoporosis medication type.

Medication (First Medication Used)	Insurance Issue, *n* (%)	No Insurance Issue, *n* (%)	*p*-Value (χ^2^ Test)
Abaloparatide (Tymlos)	15 (48.4%)	16 (51.6%)	0.1192
Oral Bisphosphonate (Fosamax, Boniva)	1 (50.0%)	1 (50.0%)	1
Intravenous Bisphosphonates (Reclast)	4 (50.0%)	4 (50.0%)	0.5905
Denosumab (Prolia)	5 (15.2%)	28 (84.8%)	**0.0124**
Other/Not started	0 (0.0%)	1 (100.0%)	1
Raloxifene (Evista)	0 (0.0%)	2 (100.0%)	0.7667
Romosozumab (Evenity)	17 (35.4%)	31 (64.6%)	1
Teriparatide (Forteo)	9 (42.9%)	12 (57.1%)	0.5646

**Table 17:** Incidence of hardware failure and DXA T-scores following treatment.

Hardware Failure, *n* (% of total cohort)	20 (11.5)
Screw Loosening	12 (6.9)
Screw Pullout	3 (1.7)
Screw Breaking	4 (2.3)
Rod Breaking	4 (2.3)
Interbody Subsidence	1 (0.6)
Hardware Failure by Diagnosis, *n* (% of diagnosis)	
Normal Bone Health	1 (20)
Osteopenia	5 (10.6)
Osteoporosis	11 (11.2)
Severe Osteoporosis	3 (12.5)
Revision Surgeries	10 (5.7)
Average Follow-Up, mean ± SD	2.9 ± 1.7 years
Number of Patients with DXA Follow-Up, *n* (%)	80 (46.0%)
Lowest DXA Scan T-score, prior to treatment, mean ± SD	−2.5 ± 1
Lowest DXA Scan T-score, after treatment, mean ± SD	−2.2 ± 1.2
Number of Patients with Improved DXA T-score, *n* (%)	48 (60.0%)
Average Improvement in DXA T-score	0.6 ± 0.5

## Data Availability

The de-identified patient data supporting the findings of this study were collected and stored in a secure, institutional REDCap database. The data are not publicly available due to institutional policy and patient privacy restrictions (HIPAA). However, the datasets generated and analyzed during the current study are available from the corresponding author upon reasonable request and with approval from the Institutional Review Board (IRB) and Data Use Agreement execution.

## Abbreviations

The following abbreviations are used in this manuscript:

ACDF	Anterior Cervical Discectomy and Fusion
ALIF	Anterior Lumbar Interbody Fusion
BHC	Bone Health Clinic
BMI	Body Mass Index
DXA	Dual-energy X-ray Absorptiometry
FLS	Fracture Liaison Service
LLIF	Lateral Lumbar Interbody Fusion
NOF	National Osteoporosis Foundation
PCDF	Posterior Cervical Discectomy and Fusion
PLIF	Posterior Lumbar Interbody Fusion
PSF	Posterior Spinal Fusion
TLIF	Transforaminal Lumbar Interbody Fusion
XLIF	Extreme Lateral Interbody Fusion
